# Causal effects of tea intake on multiple types of fractures: A two-sample Mendelian randomization study

**DOI:** 10.1097/MD.0000000000033542

**Published:** 2023-06-02

**Authors:** Bin Pu, Peng Gu, Lieliang Luo, Dan Yue, Qiao Xin, Zhanpeng Zeng, Xiaohui Zheng, WeiDong Luo

**Affiliations:** a Guangzhou University of Chinese Medicine, Guangzhou, China; b Southwest Medical University, Luzhou, China; c Jiangxi University of Traditional Chinese Medicine, Nanchang, China; d The First Affiliated Hospital, Guangzhou University of Chinese Medicine, Guangzhou, China.

**Keywords:** bone metabolism, fracture, genome-wide association studies, Mendelian randomization, tea intake

## Abstract

Fracture is a global public health disease. Bone health and fracture risk have become the focus of public and scientific attention. Observational studies have reported that tea consumption is associated with fracture risk, but the results are inconsistent. The present study used 2-sample Mendelian randomization (MR) analysis. The inverse variance weighted method, employing genetic data from UK Biobank (447,485 cases) of tea intake and UK Biobank (Genome-wide association study Round 2) project (361,194 cases) of fractures, was performed to estimate the causal relationship between tea intake and multiple types of fractures. The inverse variance weighted indicated no causal effects of tea consumption on fractures of the skull and face, shoulder and upper arm, hand and wrist, femur, calf, and ankle (odds ratio = 1.000, 1.000, 1.002, 0.997, 0.998; *P* = .881, 0.857, 0.339, 0.054, 0.569, respectively). Consistent results were also found in MR-Egger, weighted median, and weighted mode. Our research provided evidence that tea consumption is unlikely to affect the incidence of fractures.

## 1. Introduction

Fracture is usually defined as the interruption of bone integrity and continuity, which can occur in any part of the body, among which the femur (hip), vertebra (spine), and distal radius (wrist) are the most common.^[1]^ With the increasing aging trend of the global population and improving living and medical conditions, bone health and fracture risk have become the focus of public and scientific attention.^[2]^

Numerous studies have shown that fracture is closely related to the ethnic group, region, age, occupation, lifestyle, diet, and disease.^[3]^ Among them, the influence of dietary factors cannot be ignored, including coffee intake,^[4]^ alcohol consumption,^[5]^ dietary protein intake,^[6]^ etc. We are extraordinarily interested in the causality between tea consumption and fracture risk among dietary factors. Tea is the second-most consumed beverage in the world. It is reported that tea drinking has particular benefits in reducing the risk of osteoporosis,^[7]^ primary prevention of cardiovascular diseases,^[8]^ and blood glucose control.^[9]^ Previous studies have investigated the relationship between tea consumption and fracture risk,^[10–13]^ but the results vary among populations in different countries and regions.

Mendelian randomization (MR) is considered a method comparable to randomized controlled studies (RCT).^[14]^ By using large-scale genome-wide association study (GWAS) data, genetic variation is used as an instrumental variable (IV) to derive the causal relationship between outcome and exposure.^[15]^ This method can effectively avoid the confounding bias and reverse causal bias of traditional epidemiological studies. Previous studies used MR to report the causal relationship between tea intake and obesity, stroke, type 2 diabetes, and renal function. However, no study has used MR to report the causal relationship between tea intake and fracture. Therefore, we innovatively proposed to use the MR method to explore the causal relationship between tea intake and fracture from the level of genetic variation, which is significant for preventing fracture, improving bone health, and formulating appropriate intervention measures.

## 2. Methods

### 2.1. Data source

We conducted a 2-sample MR study using aggregate data on tea intake and multiple types of fractures from different GWAS. To reduce the potential confounding bias caused by ethnic stratification, we limited the sample data to the European population.

The tea intake (phenotype code: 1488_RAW) data set was obtained from the diet questionnaire data of the British Biobank. The GWAS adjusted for sex, age, age^2^, sex × age, sex × age^2^, and the first 20 ancestry principal components. The tea intake in the dietary data began to recruit participants in 2006 and was measured in cups per day. Those with answers of <0 or >99 were excluded, and participants with > 20 were asked to reconfirm, including 447,485 samples of European descent. For more details regarding tea intake, please visit https://biobank.ctsu.ox.ac.uk/crystal/field.cgi?id=1488. We extracted aggregate statistics on the skull and facial, shoulder and upper arm, hand and wrist, femur, calf, and ankle fractures from Chapter XIX Injury, poisoning, and other consequences of external causes of UK Biobank (GWAS Round 2). This data includes 361,194 cases of fracture. Details of the fracture were obtained at https://biobank.ctsu.ox.ac.uk/crystal/field.cgi?id=41202.

### 2.2. MR analysis

MR refers to using genetic variation to estimate the causal relationship between exposure and outcome. Exposure can be anthropometric indicators, laboratory testing indicators, or other risk factors that may affect the outcome. The outcome is usually a specific disease, but not limited to disease. The genetic variation must satisfy the hypothesis of IV: closely associated with exposure; not associated with confounding factors associated with exposure-outcome; and no direct relationship between genetic variation and outcome.^[16]^

Single nucleotide polymorphism (SNP) is essential in exploring the human genome’s structure and understanding the etiology and pathological process of many diseases. Studying genotypes and diseases can simulate the relationship between environmental exposure factors and diseases. SNP (DNA sequence diversity caused by single nucleotide variation) generally represents the difference in genotypes.^[17]^ To infer the causal effects of tea intake and fracture, we calculated the ratio between the SNP effect on fracture and the SNP effect on tea intake.

The present study obtained 41 unique SNPs by selecting significant exposure of SNP (*P* < 5 × 10^−8^) and checking for linkage disequilibrium effect size outliers. We chose not to use the SNP proxy and set the minimum allele frequency (MAF) to 0.3. In addition, we coordinated the effect alleles in the exposure and result data sets, excluding all SNP with palindromes.^[18]^ SNPs with A/T or G/C alleles are defined as palindrome SNPs.^[19]^ We chose fixed effect inverse variance weighted (IVW) for causal analysis because the causal relationship between SNPs was under-dispersed, while the random effect IVW model will exaggerate the variance.^[18]^ IVW was insensitive to horizontal pleiotropy,^[20]^ so we used MR-Egger, weighted median, and weighted model to evaluate the causality between tea intake and fracture.^[21]^ Secondly, Cochran’s Q test was used to evaluate the heterogeneity among different SNP. If *P* < .05, it was defined as significant heterogeneity.^[22]^ Then the intercept term tested by the MR-Egger model was away from 0, indicating the existence of horizontal pleiotropy.^[23]^ The leave-one-out method was used for sensitivity analysis.^[24]^ Judge whether a bias was caused by weak IV on the results according to *R*^2^ and *F* value test (see Table 1 for calculation formula).^[25]^ *F* statistics > 10 indicated that weak IV deviations were unlikely.^[26]^

**Table 1:** Characteristics of single-nucleotide polymorphisms (SNPs) associated with tea consumption.

SNP	EA	Position	EAF	BETA	SE	*P*	N	*R* ^2^	*F*
rs10741694	C	11.16286183	0.63	0.015	0.0022	7.90E-12	447,485	0.0001045	47
rs10752269	A	10.12692902	0.51	0.0129	0.0021	1.30E-09	447,485	0.0000824	37
rs10764990	A	10.129152608	0.61	0.0122	0.0022	1.90E-08	447,485	0.0000706	32
rs11164870†	G	1.93552187	0.6	−0.012	0.0022	4.20E-08	447,485	0.0000671	30
rs1156588	G	2.58515375	0.21	0.0155	0.0026	2.90E-09	447,485	0.0000787	35
rs11587444	G	1.150722844	0.39	0.014	0.0022	1.00E-10	447,485	0.0000934	42
rs12591786	T	15.60902512	0.16	0.0184	0.0029	3.70E-10	447,485	0.0000878	39
rs13282783	T	8.22088975	0.29	0.0136	0.0024	7.90E-09	447,485	0.0000744	33
rs132904†	C	22.41798896	0.78	0.0166	0.0026	7.80E-11	447,485	0.0000945	42
rs141071726	A	7.17558580	0.03	0.0407	0.0068	2.20E-09	447,485	0.0000799	36
rs1453548†	A	11.59192089	0.66	0.0133	0.0022	3.00E-09	447,485	0.0000786	35
rs1481012	G	4.89039082	0.11	0.0262	0.0034	5.30E-15	447,485	0.0001366	61
rs149805207*	G	6.137095269	0.01	0.0719	0.0126	1.10E-08	447,485	0.0000730	33
rs17245213	A	11.1679769	0.21	0.0146	0.0026	2.00E-08	447,485	0.0000704	32
rs17576658	A	13.100272019	0.25	0.0135	0.0025	4.10E-08	447,485	0.0000673	30
rs17685	A	7.75616105	0.28	0.0231	0.0024	1.60E-22	447,485	0.0002131	95
rs2117137	G	3.89525505	0.41	0.013	0.0022	1.70E-09	447,485	0.0000812	36
rs2273447†	T	20.62900120	0.2	0.0175	0.0026	3.30E-11	447,485	0.0000983	44
rs2279844	A	17.40819809	0.38	−0.012	0.0022	4.00E-08	447,485	0.0000674	30
rs2351187	A	10.86850616	0.32	0.0129	0.0023	1.60E-08	447,485	0.0000714	32
rs2472297	T	15.75027880	0.26	0.0533	0.0024	2.30E-109	447,485	0.0011019	494
rs2478875	G	6.51283110	0.21	0.0219	0.0026	5.10E-17	447,485	0.0001571	70
rs2645929	G	13.56444529	0.81	−0.015	0.0027	3.50E-08	447,485	0.0000680	30
rs2783129†	G	13.80168720	0.48	0.0117	0.0021	3.80E-08	447,485	0.0000676	30
rs34619	A	5.60465365	0.43	0.0117	0.0021	4.30E-08	447,485	0.0000671	30
rs4410790	C	7.17284577	0.63	0.0406	0.0022	3.40E-76	447,485	0.0007621	341
rs4808193	C	19.19410622	0.34	0.0151	0.0022	1.70E-11	447,485	0.0001011	45
rs4817505	C	21.34343828	0.39	0.0151	0.0022	4.20E-12	447,485	0.0001073	48
rs56188862	C	1.174189269	0.39	0.0158	0.0022	4.30E-13	447,485	0.0001173	52
rs56348300†	G	9.7054124	0.18	0.0159	0.0027	6.10E-09	447,485	0.0000755	34
rs57462170	A	3.50239803	0.11	0.0192	0.0034	1.90E-08	447,485	0.0000707	32
rs57631352	G	19.4338173	0.3	0.0131	0.0023	1.70E-08	447,485	0.0000712	32
rs6829	T	13.111531264	0.6	0.0119	0.0022	3.70E-08	447,485	0.0000677	30
rs713598†	G	7.141673345	0.4	0.0134	0.0022	5.20E-10	447,485	0.0000862	39
rs72797284	G	5.152031650	0.27	0.0171	0.0024	7.00E-13	447,485	0.0001152	52
rs7757102	G	6.137222671	0.56	0.0118	0.0021	3.10E-08	447,485	0.0000684	31
rs9302428†	G	16.24717600	0.64	0.0122	0.0022	2.60E-08	447,485	0.0000692	31
rs9624470	A	22.24820268	0.58	0.0252	0.0022	1.30E-31	447,485	0.0003057	137
rs9648476	A	7.39293033	0.62	0.0125	0.0022	1.10E-08	447,485	0.0000731	33
rs977474	T	12.11284772	0.83	0.0218	0.0029	2.40E-14	447,485	0.0001300	58
rs9937354	A	16.53799847	0.42	0.0141	0.0021	4.90E-11	447,485	0.0000966	43

*P*, the significance level of tea; *R*^2^ was calculated as follows: 2*beta^2*EAF*(1-EAF)/(2*beta^2*EAF*(1-EAF) + se^2*2*N*EAF(1-EAF)). The *F* statistic for each SNP was calculated as follows: *F* = (N − 2)*R^2^/(1 − *R*^2^).

BETA = beta exposure, EA = effect allele, EAF = effect allele frequency, SE = standard error, SNP = single-nucleotide polymorphism.

* Missing of the skull and facial fractures.

† Palindromes were excluded.

All data were analyzed using *R* software v.4.0.3 and MR-base. All images were generated by GraphPad Prism 9.0.0 and Adobe Illustrator 2021. *P* < .05 indicated statistical significance (unless stated separately).

## 3. Results

### 3.1. IV selection

41 SNPs related to tea intake were selected in the MR analysis (*P* < 5 × 10^−8^, through linkage disequilibrium analysis, *r*^2^ = 0.0001, kb = 10,000). Among them, 8 SNPs were removed because of being palindromes. Finally, 33 SNPs related to tea intake were selected to perform the following MR analysis (skull and facial fractures:32 SNPs, missing rs149805207) (Table 1). The F statistics of SNPs were all greater than 10 (mean value = 67, *F* min = 30, *F*max = 494).

### 3.2. Two-sample MR analysis

**Figure 1.**
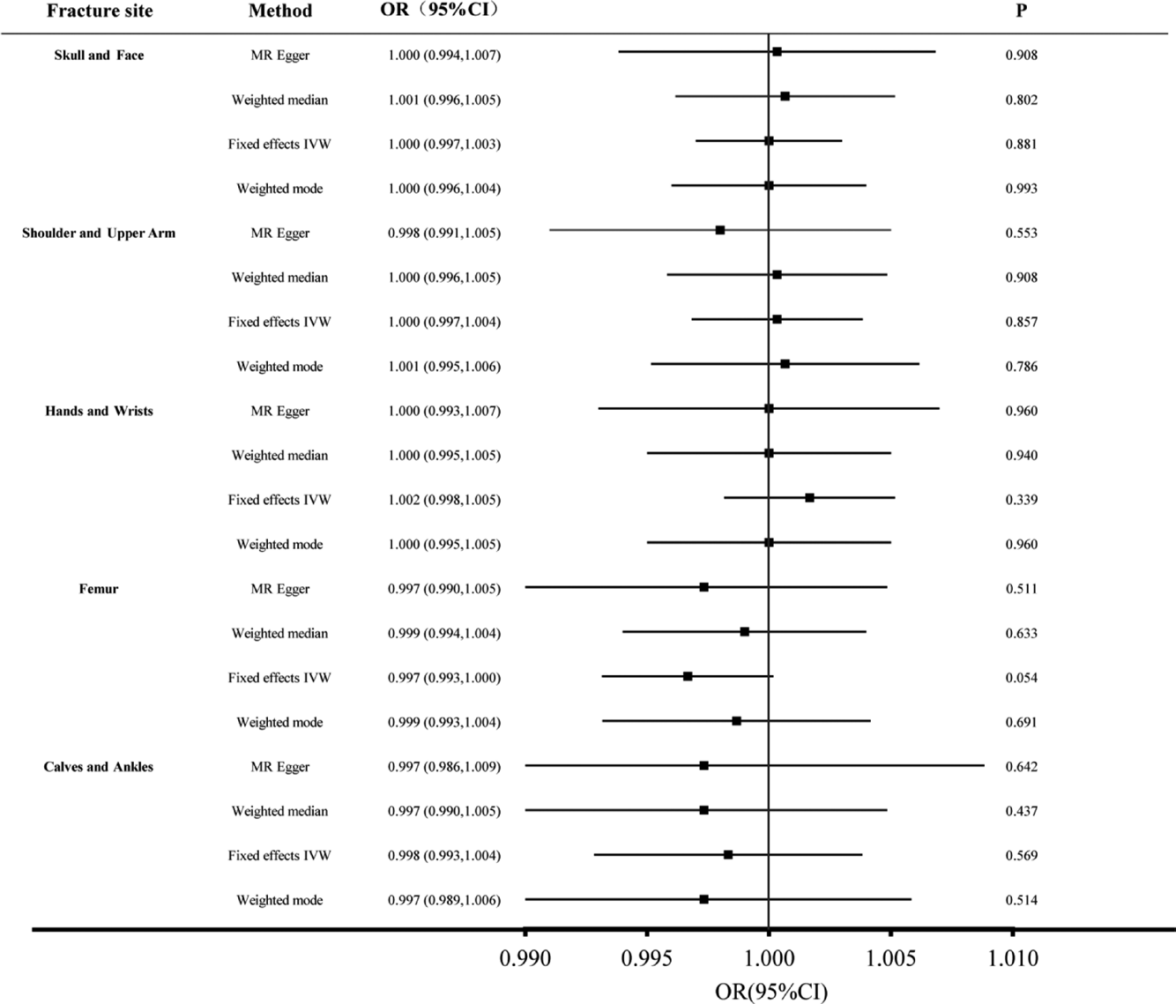
Forest plots of Mendelian randomization (MR) study using genetically predicted tea intake with multiple types of fractures. Inverse variance weighted (IVW), MR-Egger, weighted median, and weighted mode were used in this study.

**Figure 2.**
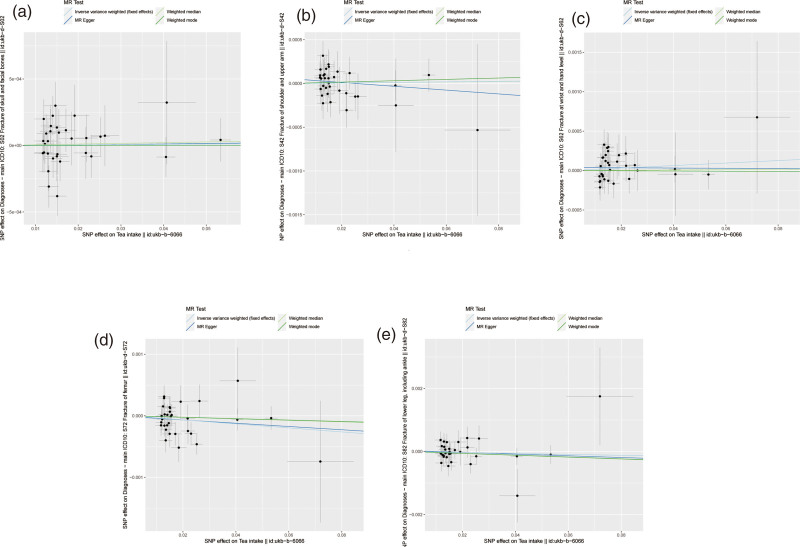
The scatter plot for MR analyses of causal associations between each tea intake SNP and multiple types of fractures. MR = Mendelian randomization, SNP = single nucleotide polymorphism.

The Fixed effect IVW analysis indicated that the genetic prediction of drinking an extra cup of tea a day did not affect fracture risk. The OR value of increased tea intake for skull and facial fractures was 1.000 (95% CI, 0.997–1.003), shoulder and upper arm fractures were 1.000 (95% CI, 0.997–1.004), hand and wrist fractures were 1.002 (95% CI, 0.998–1.005), femur fractures were 0.997 (95% CI, 0.993–1.000), calf and ankle fractures were 0.998 (95% CI, 0.993–1.004) (Fig. 1). Similar results were observed in MR-Egger, weighted median, and weighted mode (Fig. 1). The scatter plot of these results is shown in Figure 2. Cochran’s Q test indicated no significant heterogeneity (Table 2), and the visualization results were shown in the funnel diagram (Figure S1, Supplemental Digital Content, http://links.lww.com/MD/I811). The MR-Egger test did not detect horizontal pleiotropy (Table 2). The left-out method showed that the comprehensive effect of SNP was not changed or reversed one by one (Figure S2, Supplemental Digital Content, http://links.lww.com/MD/I812). The forest diagram of the tea intake of each SNP and different fracture estimates is shown in Figure S3, Supplemental Digital Content, http://links.lww.com/MD/I813.

**Table 2:** Results of horizontal pleiotropy and heterogeneity statistics.

Outcome	Cochran		MR-Egger	
	Q	*P*	Intercept	*P*
Skull and face	26.91	.63	−0.0000034	.96
Shoulder and upper arm	18.53	.96	0.000055	.45
Hands and wrists	21.06	.91	0.000039	.58
Femur	36.59	.23	−0.000014	.86
Calves and ankles	21.59	.90	0.000026	.82

MR = Mendelian randomization.

## 4. Discussion

In this study, we used 2 samples of MR to investigate the causal relationship between tea intake and fracture. It revealed no causal relationship between genetically predicted tea intake and fracture risk among 361,194 fracture cases and 447,485 tea intake participants in the sample, supporting the results of most but not all observational studies.

Numerous studies have reported the relationship between tea intake and fracture risk. Da Veiga et al^[10]^ studied the fracture risk of yerba mate intake in 95 postmenopausal women in southern Brazil. They found that yerba mate intake did not affect fracture but bone metabolism. Dai et al^[27]^ found no relationship between tea intake and hip fractures in a prospective cohort of 63,257 Singaporean-based people aged 45 to 74. Hallström et al^[28]^ conducted a cohort study of 31,527 Swedish women aged 40 to 76 years and found that tea consumption was unrelated to fracture risk. Tavani et al^[29]^ found no association between tea intake in 279 women with hip fractures in northern Italy. In a case-control study on the effect of diet on the risk of postmenopausal hip and wrist fractures, Kreiger et al^[30]^ revealed that tea intake was not associated with fracture risk (hip and wrist). These findings are in accord with our MR analysis. However, Myers et al^[12]^ found that higher black tea and specific classes of flavonoid intake were associated with a lower risk of fractures in older women. A prospective study of 1027 Western Australian women by Devine et al^[31]^ found that tea drinking was associated with hip bone protection in older women. Shen et al^[32]^ studied tea consumption and fracture risk among 453,625 Chinese adults and found that daily tea drinkers had a lower risk of fracture hospitalization than non-tea drinkers over the previous 12 months. In addition, Johnel et al found that tea intake is one of the risk factors for hip fracture in European people over 50 years old.^[33,34]^ Taken together, the protective effects of tea consumption on fracture risk were mainly detected in studies conducted in Asian and Oceanian countries. However, the results in European populations were irrelevant or negative.

The inconsistent results of studies may be related to the influence of tea components on bone metabolism, which explains the microscopic mechanism of the 2 effects from the perspective of bone mineral density and bone strength. Tea is rich in caffeine, polyphenols, flavonoids, alkaloids, etc. Their effects on bones can be divided into 2 categories. Protective effect of tea components on bone. Polyphenols can enhance the ability of antioxidation and reduce oxidative stress damage, which has a beneficial effect on bone metabolism.^[35–37]^ Flavonoids can affect bone health by increasing osteogenic gene expression, stimulating osteogenesis, and improving bone marker activity.^[38]^ Similar results appeared in Chen et al’s study on the effect of (-) -epigallocatechin-3-gallate (EGCG) on the osteogenic function of mouse bone marrow mesenchymal stem cells.^[12]^ In addition, a study of postmenopausal women found that serum NOx levels increased significantly in women who drank yerba mate.^[10]^ High levels of NO seemed to stimulate osteoprotegerin and inhibit RANK-RANKL binding from reducing bone resorption, acting as an estrogen mediator.^[39]^ Other studies also found that drinking tea may increase bone density by affecting fluoride levels in the body.^[28,40]^ The damaging effect of tea components on bone. The caffeine in tea hurts bones. Experimental studies have proved that high-dose caffeine intake can affect the bone development of growing rats.^[41,42]^ The mechanism may be related to promoting calcium excretion in urine and feces,^[43,44]^ resulting in a negative calcium balance, which reduces bone density and bone strength. This possible mechanism has also been verified in several studies on caffeine intake and human metabolism.^[45,46]^ Another interesting mechanism is that tea and flavonoids affect bone health by affecting cardiovascular health,^[12]^ a conjecture that needs to be confirmed by more studies. In addition, tea is a stimulant beverage containing many alkaloids such as caffeine, theobromine, and theophylline. It may promote the excitement of the motor nervous system, which increases the risk of falls and leads to fractures.

RCT is recognized as the gold standard for studying whether interventions affect health outcomes. However, RCT is often expensive, impractical, or has a high failure rate.^[47]^ MR is a statistical method that uses a genetic variation to simulate RCT, infer the causal relationship between phenotypes, and understand the etiology of the disease process.^[18]^ Compared with traditional epidemiological studies, MR studies take advantage of the essentially unmodifiable nature of the germline genome, which is not susceptible to reverse causal bias and confounding factors.^[48]^ Finally, 33 SNP were selected (32 SNP for skull and facial fractures), and IVW, MR-Egger, weighted median, and weighted model were used to analyze the causality between the 2 samples. The results still showed that tea intake had no causal relationship or effect on fracture risk. Thus, we speculate that the interaction of these 2 mechanisms may offset the impact of tea intake on bones. In addition, we believe that the human body, as an organic whole, has a robust homeostasis regulation system, and the protective and damaging effects of tea intake on the body are insufficient to cause tissue and organ damage. As a result, a causal relationship between tea intake and fracture risk is unlikely.

A major strength of our study is that it is the first time to use the 2-sample MR to analyze the causal relationship between tea intake and fracture risk. The method overcomes the inherent effects of residual confusion, reverse causality bias, and measurement errors in traditional epidemiology. Another advantage is that we use multiple types of fractures as outcome variables to avoid the partial generalization caused by a single site. However, this study has some inevitable limitations. Firstly, the results did not apply to other races and regions due to deviations from the data limited to European populations. Secondly, the tea varieties and production methods are multifarious, which is extraordinarily significant in exploring the causal relationship between tea intake and exposure genes. Further analysis of tea intake and human health should consider the overall impact of these factors.

## 5. Conclusion

In conclusion, our study did not support a causal association between tea consumption and the incidence of fractures. However, it is necessary to consider valid information on tea consumption, including the tea varieties and production methods, in further analysis of tea consumption and the incidence of fractures.

## Author contributions

**Conceptualization:** Peng Gu.

**Data curation:** XiaoHui Zheng.

**Funding acquisition:** WeiDong Luo.

**Investigation:** WeiDong Luo.

**Methodology:** Dan Yue.

**Resources:** Dan Yue, Qiao Xin.

**Software:** Dan Yue, Qiao Xin, ZhanPeng Zeng.

**Validation:** Peng Gu, XiaoHui Zheng.

**Visualization:** ZhanPeng Zeng.

**Writing – original draft:** Bin Pu, Peng Gu.

**Writing – review & editing:** Bin Pu.

## Supplementary Material






