# Supplementary material for: Causal effects of tea intake on multiple types of fractures: A two-sample Mendelian randomization study
Source: Medicine (Baltimore). 2023 Jun 2;102(22):e33542. doi: 10.1097/MD.0000000000033542 (PMC10238023; doi:10.1097/MD.0000000000033542)

Supplementary Materials

Supplementary Figure2. Leave-one-out sensitivity analysis for multiple types of fractures using SNP-associated tea intake.

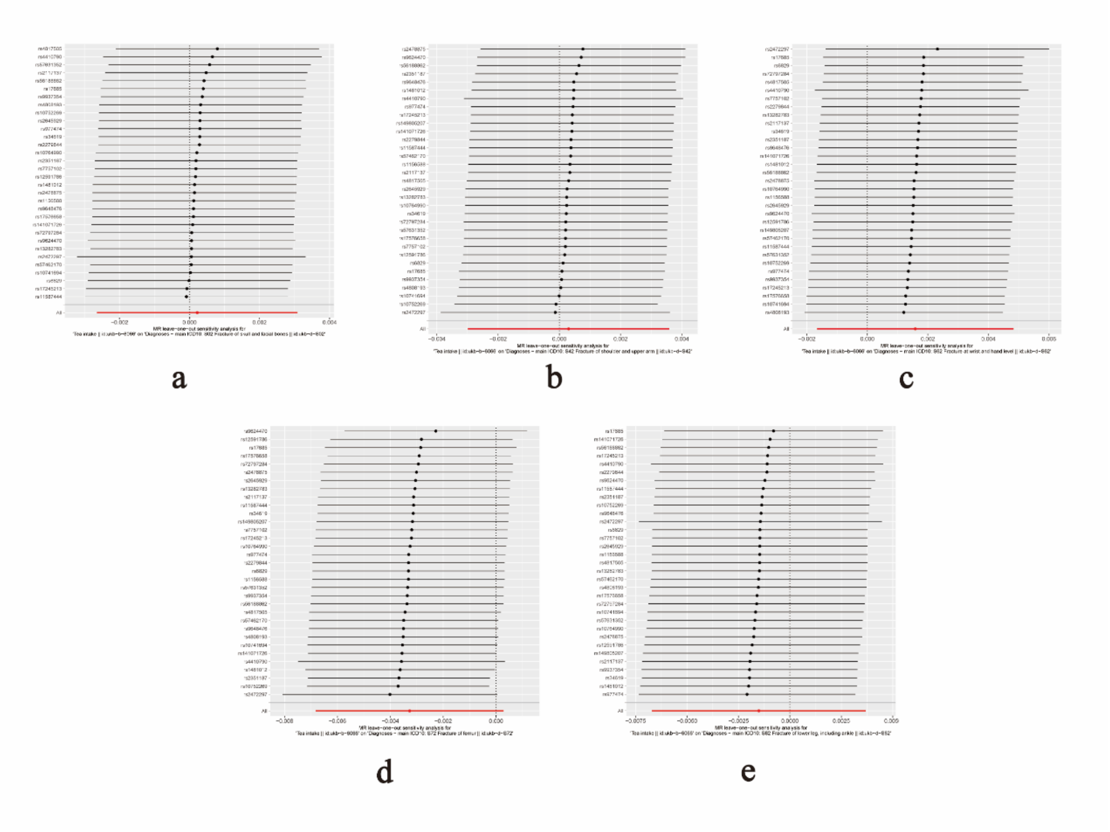

Supplement: Supplementary file 2 [file medi-102-e33542-s002.pdf]
