# Supplementary material for: Causal effects of tea intake on multiple types of fractures: A two-sample Mendelian randomization study
Source: Medicine (Baltimore). 2023 Jun 2;102(22):e33542. doi: 10.1097/MD.0000000000033542 (PMC10238023; doi:10.1097/MD.0000000000033542)

# *Supplementary Materials*

**Supplementary Figure3.** The forest plot for MR analyses of causal associations between each tea intake SNP and multiple types of fractures.

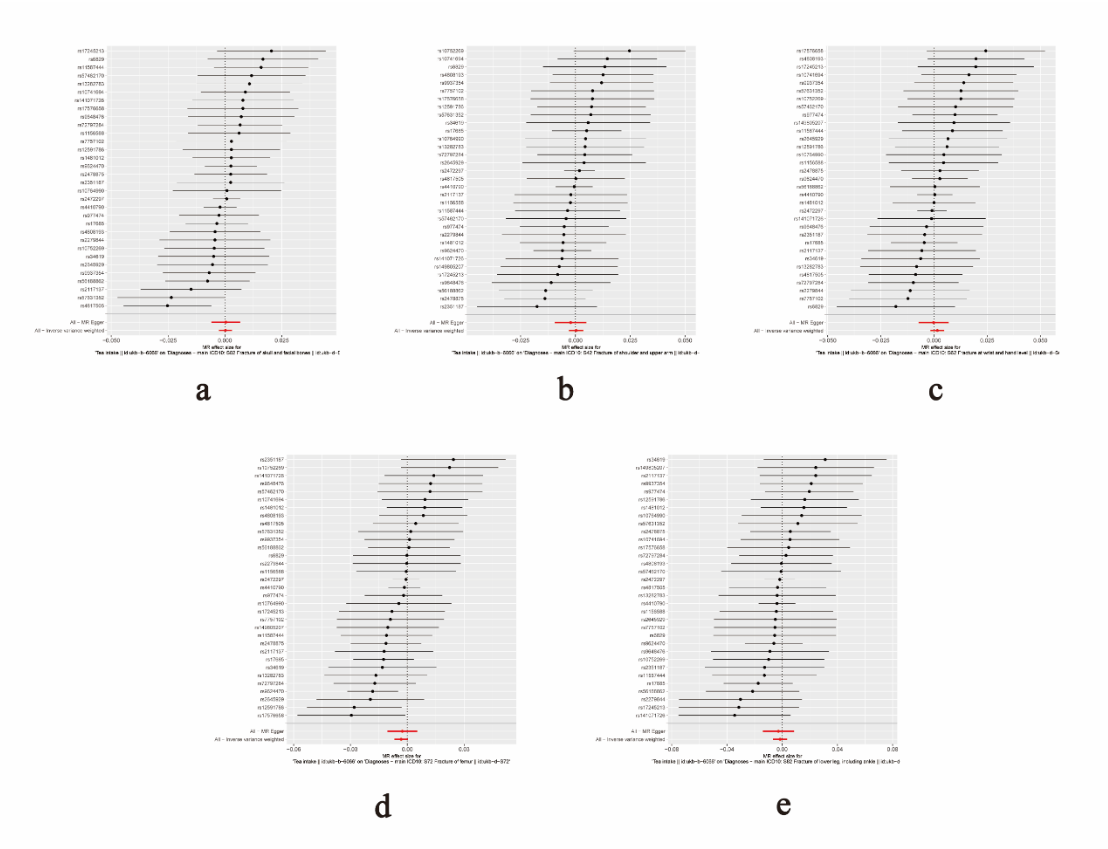

Supplement: Supplementary file 3 [file medi-102-e33542-s003.pdf]
